# The CUGBP2 Splicing Factor Regulates an Ensemble of Branchpoints from Perimeter Binding Sites with Implications for Autoregulation

**DOI:** 10.1371/journal.pgen.1000595

**Published:** 2009-08-14

**Authors:** Jill A. Dembowski, Paula J. Grabowski

**Affiliations:** Department of Biological Sciences, University of Pittsburgh, Pittsburgh, Pennsylvania, United States of America; University of California San Francisco, United States of America

## Abstract

Alternative pre-mRNA splicing adjusts the transcriptional output of the genome by generating related mRNAs from a single primary transcript, thereby expanding protein diversity. A fundamental unanswered question is how splicing factors achieve specificity in the selection of target substrates despite the recognition of information-poor sequence motifs. The CUGBP2 splicing regulator plays a key role in the brain region-specific silencing of the NI exon of the NMDA R1 receptor. However, the sequence motifs utilized by this factor for specific target exon selection and its role in splicing silencing are not understood. Here, we use chemical modification footprinting to map the contact sites of CUGBP2 to GU-rich motifs closely positioned at the boundaries of the branch sites of the NI exon, and we demonstrate a mechanistic role for this specific arrangement of motifs for the regulation of branchpoint formation. General support for a branch site-perimeter–binding model is indicated by the identification of a group of novel target exons with a similar configuration of motifs that are silenced by CUGBP2. These results reveal an autoregulatory role for CUGBP2 as indicated by its direct interaction with functionally significant RNA motifs surrounding the branch sites upstream of exon 6 of the CUGBP2 transcript itself. The perimeter-binding model explains how CUGBP2 can effectively embrace the branch site region to achieve the specificity needed for the selection of exon targets and the fine-tuning of alternative splicing patterns.

## Author Summary

Alternative splicing is a precisely controlled process that determines whether an exon will be included or skipped in the mature mRNA transcript. Factors that control alternative splicing bind to RNA sequence motifs in the exon or flanking introns and guide tissue and developmental specific splicing events. CUGBP2 is a dual functional regulator of alternative splicing that can cause inclusion or skipping of a target exon, depending on the context of its binding motifs. Previously, the mechanisms of regulation by this protein and the positional significance of its target motifs have not been characterized. In this study, the authors dissected the mechanism of exon skipping by CUGBP2 and demonstrate that a specific configuration of motifs at the perimeters of a functional reference point are intimately involved in this event. Furthermore, this mechanism of regulation is shown to have general significance because novel CUGBP2 target exons contain a similar arrangement of motifs. The most interesting of this group is an exon within the CUGBP2 transcript itself. This study underscores the importance of a functional reference point in the specificity of regulation by an alternative splicing factor and reveals a novel autoregulatory role for CUGBP2.

## Introduction

Alternative pre-mRNA splicing is prevalent throughout vertebrate genomes where an individual gene can be diversified into hundreds or even thousands of related mRNA isoforms [1],[2]. Functional consequences of alternative splicing can involve changes to a subset of the protein's biochemical properties or subcellular localization. These are powerful mechanisms used to regulate protein functions across different cell types, during development, or in response to extracellular signals [3],[4]. One of the major challenges in postgenome biology is to understand how alternative splicing, which involves a high degree of inherent flexibility, can achieve the specificity needed to select the correct set of target transcripts for regulation.

The spliceosome is the functional context for regulation, since this is the macromolecular machinery that guides intron removal and exon joining. It is assembled from the dynamic associations of five small nuclear ribonucleoprotein particles (snRNPs) and hundreds of accessory factors [5],[6]. Initially, U1 snRNP and U2AF (U2 snRNP auxiliary factor) recognize the 5′ and 3′ splice sites of the exon, respectively, and U2 snRNP base pairs with the branch site region thereby designating the adenosine to be used as the branchpoint. The association of U456 tri-snRNP and various RNA rearrangements then activate the first step of catalysis, which generates the 5′ exon and lariat intron-3′ exon intermediate. As catalysis advances to the second step, the lariat intron is excised and the 5′ and 3′ exons are ligated. The overall pattern of exon inclusion/skipping depends on the ability of the spliceosome to recognize each splice site signal, which is a reflection of the inherent strength of the site as well as the regulatory effects of splicing factors acting from sequence motifs nearby [2],[7]. Exon definition, which involves the interactions of U1 snRNP bound to the 5′ splice site and U2AF bound to the 3′ splice site across an exon is a particularly sensitive mechanism to specify alternative splicing patterns [8],[9].

Two families of RNA binding proteins known to regulate alternative splicing by direct recognition of RNA sequence motifs include the arginine-serine rich (SR) and hnRNP splicing factors [10],[11]. SR splicing factors most commonly recognize exonic splicing enhancers, whereas hnRNP proteins recognize intronic or exonic splicing silencers and enhancers. These regulatory motifs are typically short and degenerate making it difficult to reliably predict the target exons of a splicing factor based upon sequence inspection alone. CUG Binding Protein 2, or CUGBP2 (also called NAPOR, CELF-2, ETR-3, or BRUNOL3) is a member of the larger family of CUGBP and ETR-3-like (CELF) RNA binding proteins, which have been shown to regulate alternative splicing through UG-rich motifs in accordance with their tissue-specific expression patterns [12],[13]. CELF proteins have been shown to play important roles in heart development, whereas their misregulation has been implicated in the pathogenesis of myotonic dystrophy [14]–[16].

We previously reported a close correlation between the distribution of protein expression patterns of CUGBP2 (called NAPOR in the previous study) and splicing patterns of the NI and CI cassette exons of the NMDA R1 receptor transcript (*GRIN1* gene) in the rat brain [17]. In particular, high levels of CUGBP2 protein in the forebrain were associated with skipping of the NI exon and inclusion of the CI exon, and these splicing patterns reversed in the hindbrain where CUGBP2 was deficient. *In vivo* splicing reporter assays confirmed dual functional roles for CUGBP2 as a splicing silencer of the NI exon and as an enhancer of the CI exon. These dual roles are thought to be important in directing the brain region-specific distribution of GRIN1 mRNA isoforms for fine-tuning of receptor functions at the synapse [17]. Additional splicing factors have been shown to exhibit dual roles in enhancement and silencing depending upon the context of the target exons, but these mechanisms are, at present, poorly understood [18],[19].

In this study, we focus on the silencing face of CUGBP2's dual character to understand how it recognizes the NI target exon and the mechanism used for splicing silencing. A variety of intronic UG-rich motifs can be found within several hundred nucleotides of the NI and CI exons by sequence inspection, but functionally significant motifs in these regions have not yet been identified. We initially used a chemical-based RNA footprinting approach to detect RNA-protein interactions at nucleotide-level resolution. Here we identify the direct contact sites of CUGBP2 in the 3′ splice site region of the NI exon, and establish that this arrangement of binding sites plays a mechanistic role in silencing a group of branch sites in between. We show the significance of this mechanism by demonstrating its involvement in the regulation of other skipped exons, most notably exon 6 of the CUGBP2 transcript itself.

## Results

### CUGBP2 contacts conserved UGUGU and GU motifs surrounding the predicted branch site region upstream from the NI cassette exon

**Figure 1:**
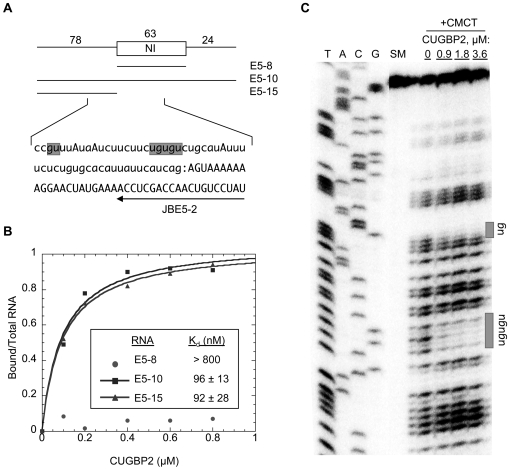
CUGBP2 contacts multiple motifs in the intron upstream from the NI cassette exon. (A) Schematic of the NI exon (rectangle) and flanking introns (lines) are shown with corresponding nucleotide lengths (numbers, top). RNA substrates (E5-8, E5-10, and E5-15) used for filter binding assays are indicated below. Sequence analyzed by footprinting using primer JBE5-2 (arrow) includes the intron (lower case) and exon (uppercase) region of the 3′ splice site (colon). Shaded sequences represent regions protected from chemical modification by CUGBP2. Individual adenosines in uppercase represent branchpoints determined by primer extension (see below, Figure 4). (B) Nitrocellulose filter binding analysis. ^32^P-labeled RNA substrates from (A) were assembled with purified recombinant CUGBP2 protein and separated into protein bound and unbound fractions. Representative graphs for each RNA substrate are shown. Inset: K_d_ values were calculated as the average of three experiments; ±, standard deviation. (C) RNA footprint analysis. E5-10 RNA was chemically modified with CMCT in the presence or absence of purified CUGBP2 protein. Modified positions were detected by primer extension (lanes +CMCT), in reference to a sequencing ladder generated from plasmid, E5-10 (lanes T,A,C,G). Shaded rectangles at right represent protected regions highlighted in the sequence shown in (A). Primer extension of starting material without modification is shown (lane SM).

A silencing role for CUGBP2 was shown for the NI exon of the GRIN1 transcript in a previous study but the mechanism of silencing was not characterized [17]. To gain insight into the mechanism, we sought to extend this analysis to identify the sequence and spatial arrangement of motifs associated with direct binding of CUGBP2 and silencing of the NI exon. A nitrocellulose filter binding assay was used initially to locate the RNA region involved in stable binding by CUGBP2. Bound/total RNA was plotted as a function of increasing protein concentration and data were fit to a hyperbola to estimate the dissociation constants (K_d_) for binding to individual RNA transcripts. These RNA substrates included the NI exon, the NI exon and flanking introns, and the upstream intron, substrates E5-8, E5-10, and E5-15, respectively (Figure 1A). CUGBP2 was found to bind to the E5-10 and E5-15 substrates containing the upstream intron region with apparent dissociation constants in the nanomolar range (96 and 92 nM K_d_ values), but not to the E5-8 substrate containing the exon alone (Figure 1B). Because the downstream intron region is not a common feature of the high affinity binding substrates, this region, as well as the exon, must be dispensable for high affinity binding.

To identify the specific nucleotides contacted by CUGBP2, we next carried out chemical modification footprinting with the E5-10 substrate, which contains the high affinity region identified by filter binding. CMCT modification at the N3 position of uracil and the N1 position of guanine causes termination of reverse transcription initiated at a downstream primer [20]. This chemical was chosen for footprinting because of the binding preference of human ETR-3 for (C)UG-rich motifs as indicated by an iterative selection procedure [13]. A representative footprint of the region upstream from the NI exon is shown (Figure 1C). These results reveal that a core (UGUGU) and upstream flanking (GU) motif are protected by CUGBP2 in a dose-dependent manner. Regions within the NI exon and in the downstream intron were also subjected to RNA footprinting with CUGBP2, but no additional binding sites were detected in agreement with the filter binding experiments.

To verify the specificity of the assay, additional footprinting reactions were carried out with purified splicing factors U2AF and PTB. Protected regions were distinct from those observed for CUGBP2 and consistent with the known RNA binding specificities of these factors (Figure S1, lanes 1–8). We also demonstrate that PTB can compete with CUGBP2 for binding to this region of RNA. That is, when PTB binds to its cognate sites which overlap with the core UGUGU motif, the pattern of CUGBP2 protection is lost from not only the core motif but also the upstream GU motif, suggesting that one CUGBP2 protein simultaneously contacts both of these sites (Figure S1, lanes 9–12). Notably, the core and flanking motifs protected by CUGBP2 are located at the boundaries of the predicted branch site region with the nucleotides protected by U2AF also within these boundaries. Thus, these results together with the high sequence conservation of the motifs (100%) across human, rat, mouse, fruit fly, and chicken genomes, support their involvement in the mechanism of silencing.

### Sequence motifs identified by RNA footprinting are functionally associated with exon silencing

**Figure 2:**
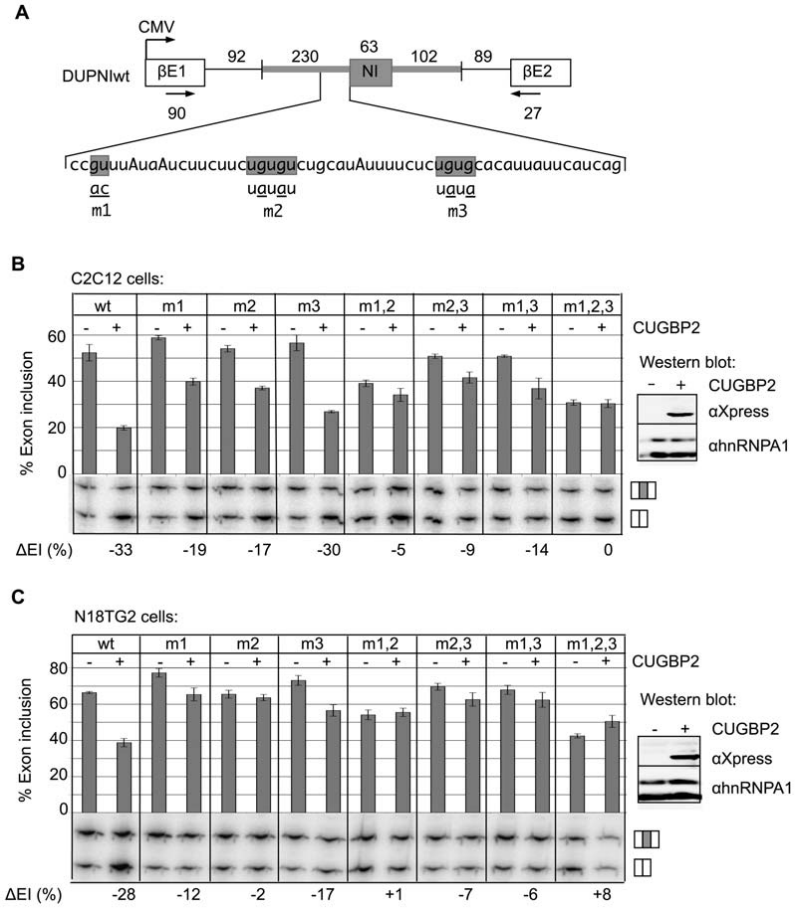
The UGUGU core motif and flanking GU dinucleotides are functionally important for CUGBP2 silencing. (A) Schematic of wild type and mutant versions of the DUPNI splicing reporter. The NI cassette exon (shaded rectangle) and regions of the native flanking introns (shaded lines) were inserted between β-globin exons βE1 and βE2 downstream of a CMV promoter. Nucleotide lengths in base pairs are indicated above and below schematic. Sequence shows an expanded view of the NI 3′ splice site region with mutations m1, m2, and m3 indicated by underscores below the shaded regions. Arrows indicate the location of primers used for RT-PCR amplification of exon included and skipped mRNAs. (B) Splicing reporter expression in C2C12 cells. Splicing reporters with no mutation (wt), with single (m1, m2, m3), or combined (m1,2, m2,3, m1,3, m1,2,3) mutations were expressed with vector backbone control (−) or with pcDNA4/CUGBP2 expression vector (+). The gel panel is a representative polyacrylamide gel image with the top band corresponding to the exon included and the bottom band corresponding the exon skipped mRNA. The bar graph shows the percent exon inclusion as an average of three separate experiments. The change in percent exon inclusion (ΔEI) as a function of CUGBP2 expression is shown below the gel panel. Inset: Western blot analysis was used to verify Xpress-tagged CUGBP2 expression; endogenous hnRNPA1 was a loading control. (C) Experiments were as in (B) except N18TG2 cells were used.

In order to determine whether nucleotides in contact with CUGBP2 upstream from the NI exon are necessary for its silencing role, we generated an *in vivo* splicing reporter with the NI exon and its immediate adjacent flanking introns inserted between β globin exons 1 and 2 (DUPNIwt) (Figure 2A). Mutant derivatives of this reporter contained site-directed mutations in the GU dinucleotide and UGUGU core motifs (m1 and m2 motifs, respectively) as identified by footprinting. A nearby UGUG motif (m3) was also mutated, since it showed weak protection in the footprinting assays (data not shown).

Splicing reporters were co-expressed in the presence and absence of CUGBP2 in C2C12 mouse myoblast cells or N18TG2 mouse neuroblastoma cells, which have little or no endogenous protein as shown by Western blotting (Figure S2). The change in exon inclusion value, ΔEI, was then calculated as the difference between the % exon inclusion±CUGBP2. The ΔEI value is used here as a convenient measure of the effectiveness of CUGBP2 to induce exon skipping (or silencing). While CUGBP2 expression in C2C12 cells induced exon skipping of the wild type substrate with a ΔEI value of −33% (Figure 2B, lanes wt) mutations in all three motifs eliminated silencing entirely as indicated by a ΔEI value of 0% (lanes m1,2,3). The double mutations also showed a significant reduction in the silencing effect of CUGBP2 (lanes m1,2, m2,3, m1,3). Of this group, combined mutations at positions m1 and m2 showed the smallest degree of silencing (ΔEI −5%), suggesting that these sites are intimately involved in the mechanism of action. Single mutations also showed a reduction in silencing indicative of additive effects (lanes m1, m2, m3). Similar results were observed in N18TG2 cells (Figure 2C). New to this cell type is higher basal levels of inclusion in the absence of CUGBP2 and reduced silencing by CUGBP2 on all substrates tested. This could be the consequence of the differential expression of splicing factors in these two cell lines. That is, an enhancer may act on the NI exon in N18TG2 cells and may be better able to compete with CUGBP2 when its binding sites are compromised. A good candidate enhancer is FOX because a perfect match to its enhancer element, (U)GCAUG, is located near the NI 5′ splice site in the downstream intron.

**Figure 3:**
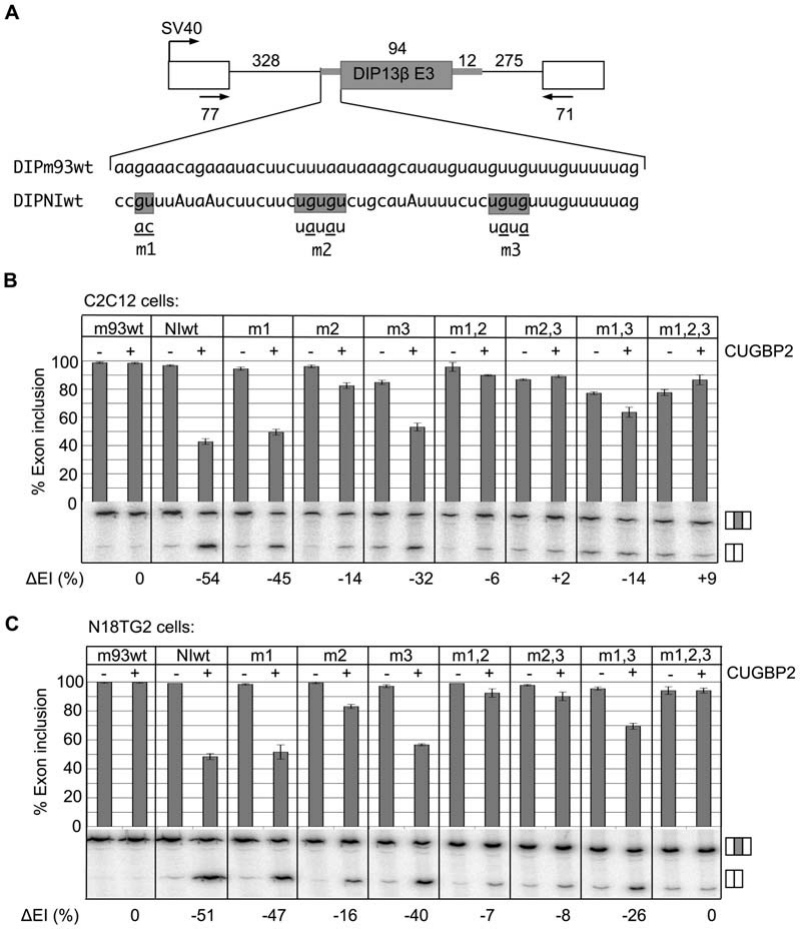
CUGBP2 silencing motifs are functionally transferable. (A) A 39 nucleotide region containing the m1, m2, and m3 silencing motifs from the NI intron (DIPNIwt) was inserted upstream from DIP13β exon 3 (middle exon) in the SIRT1 splicing reporter context. Expression was driven by the SV40 promoter. Nucleotide lengths in base pairs are indicated. Arrows indicate primers used for RT-PCR amplification; numbers below give nucleotide lengths contributing to PCR products. Individual or combinations of mutations were introduced at CUGBP2 regulatory sites as in Figure 2. Sequence of control region (DIPm93wt) corresponds to 3′ splice site region of constitutive exon DIP13β exon 3. (B) Splicing reporter plasmids were cotransfected in C2C12 cells with vector backbone control (−) or with pcDNA4/CUGBP2 expression vector (+). The graph and ΔEI calculation is as described for Figure 2. (C) Experiments were as in (B) except N18TG2 cells were used.

To further investigate the roles of the m1, m2, and m3 motifs for exon silencing by CUGBP2, we introduced a 39 nucleotide region containing the three motifs upstream of a constitutive exon in a different context (Figure 3A). Constitutive exon 3 of the DIP13β transcript was tested, since its splicing pattern is insensitive to CUGBP2 regulation, and unlike the NI exon is not under alternative splicing control. The introduction of the 39 nucleotide region conferred strong silencing by CUGBP2 (Figure 3B, lanes NIwt; ΔEI, −54%), in contrast to the parent plasmid, which was unregulated by CUGBP2 (lanes m93wt; ΔEI, ∼0%). Single and combined mutations in the m1, m2, and m3 motifs were also tested in this context (lanes m1, m2, m3, m1,2, m2,3, m1,3, and m1,2,3). Exon silencing by CUGBP2 was nearly eliminated when site-directed mutations were introduced into both the m1 and m2 positions (lanes m1,2; ΔEI, −6%). Mutations in both the m2 and m3 positions also led to a significant reduction in silencing (lanes m2,3). Thus, the general requirement for a pair of proximal CUGBP2 motifs, and the additive effects of the single mutations were verified in this context. This heterologous reporter was also tested in N18TG2 cells (Figure 3C). Here, the silencing effects of CUGBP2 were more consistent between cell lines across all mutations tested. Furthermore, compared to the NI exon, the DIP13β exon has stronger 5′ and 3′ splice sites, therefore mutations had less of an effect on the basal level of exon inclusion. Taken together, this reporter is a good system to study isolated effects of CUGBP2 on the m1, m2, and m3 motifs without indirect effects caused by other splicing factors or weak cis elements.

We also tested the silencing role of a closely related family member, CUGBP1, on wild type and mutant substrates since it is expressed in both cell lines tested (Figure S2). CUGBP1 silences the NI exon (Figure S3A, lanes DUPNI wt) with a dependence on the same motifs (lanes DUPNI m1,2,3). However, CUGBP1 silencing is much weaker in the DIPNI context (lanes DIPNI wt, ΔEI −16 compared to ΔEI −54 for CUGBP2) indicating that additional sites outside of the transferred region are necessary for strong silencing by CUGBP1. In support of this, exon 3 of the DIPNIwt reporter is included >99% of the time in N18TG2 cells (Figure 3C, lanes NIwt) despite high levels of endogenous CUGBP1 (Figure S2). Therefore, CUGBP1 shows a weaker silencing role compared to CUGBP2. As additional controls, we carried out similar experiments with overexpression of PTB or Nova, since both of these factors are known to silence the NI exon through distinct motifs (UCUU and YCAY, respectively; Y, pyrimidine). As expected, PTB and Nova were active in silencing the NI exon in the context of the DUPNIwt splicing reporter, and these effects were maintained in the presence of the m1,2,3 triple mutation (Figure S3B). Thus, the m1, m2, and m3 motifs are specific for silencing by CUGBP2.

### CUGBP2 blocks branchpoint formation between core and flanking interaction motifs

**Figure 4:**
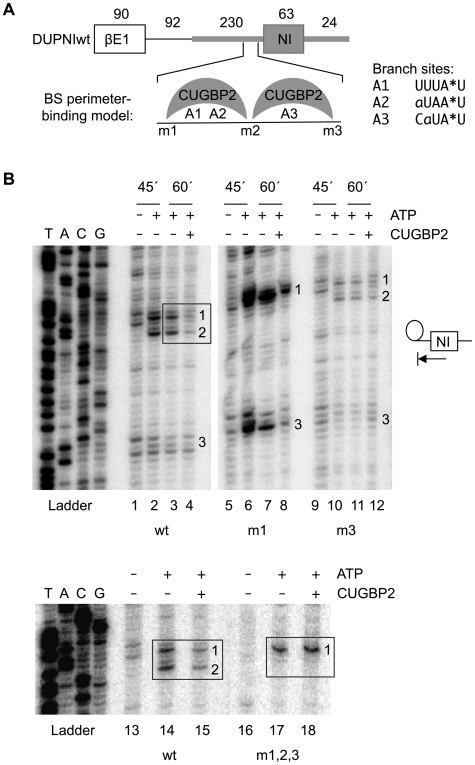
CUGBP2 blocks branchpoint formation between RNA–protein contact sites. (A) Schematic of the DUPNIwt pre-mRNA used for *in vitro* splicing assays. The NI exon and adjacent intron regions are shaded; numbers above schematic indicate nucleotide sizes. Expanded region shows relative positions of CUGBP2 motifs (m1, m2, m3) and branchpoint adenosines (A1, A2, A3) mapped in these experiments. Branch site sequences are shown at right; branchpoint adenosine, asterisk; nucleotides matching the consensus YUNAY (Y, pyrimidine; N, any nucleotide), uppercase; mismatches, lowercase. Alternate registers for CUGBP2 binding are shown schematically in the branch site (BS) perimeter-binding model. (B) Gel panels show primer extension analysis with primer, JBE5-2. Schematic at right illustrates the termination of reverse transcriptase at branchpoint positions in the assay. Branchpoint numbers on gel correspond to positions indicated on sequence in (A). A sequencing ladder is shown for the DUPNIwt plasmid (ladder). For lanes 1–12, splicing reactions containing ATP (+ATP lanes) were incubated for 45 and 60 min with (+) or without (−) 1.6 µM recombinant CUGBP2. Control reactions lacked ATP. For lanes 13–18, 60 min splicing reactions were used.

We hypothesized that CUGBP2 may function to silence the NI exon by blocking branchpoint formation in the upstream intron. Based on complementarity to U2 snRNA, two candidate branch sites, A1 and A2, are located between the m1 and m2 motifs, and a third, weaker candidate, A3, resides just downstream between the m2 and m3 motifs (Figure 4A). As a test of this hypothesis, we measured branchpoint formation for the DUPNI substrate under *in vitro* splicing conditions in the presence and absence of recombinant CUGBP2. Note that endogenous CUGBP2 levels are not detectable in Hela nuclear extracts by Western blotting with the 1H2 antibody, which is highly specific for CUGBP2 [21]. According to our model, the addition of recombinant CUGBP2 to the extract should bind and preferentially occupy motifs m1 and m2 on the wild type substrate with the resulting inhibition of one or more of the branch sites in this neighborhood. The protein can also bind in an alternate register of lower affinity by contacting a GU at the m2 site and UGUG at the m3 site. Alternately, one protein may contact all three sites simultaneously.

Branchpoints were detected by primer extension as for the experiments in Figure 1C. The results for the wild type substrate verified the use of the predicted branchpoints with a preference for A1 and A2, compared to A3 (Figure 4B, lanes 2,3). The A1 and A2 branchpoints satisfied the criteria for ATP dependence (lane 1). Primer extension of reactions following debranching showed a loss of stops at A1, A2, and A3 providing confirmation that all three of these adenosines are used as branchpoints (data not shown). Notably, branchpoint formation was inhibited when the *in vitro* splicing reactions were supplemented with recombinant CUGBP2 (Figure 4B, lane 4). An analysis of the corresponding *in vitro* splicing reactions confirmed that CUGBP2 inhibited the formation of splicing intermediates of these reactions (Figure S4, lanes wt).

We next examined the effect of the single mutation in motif m1 as a test of whether branchpoint inhibition occurs between core and flanking motifs. That is, a single mutation in m1 should permit the binding of CUGBP2 to the remaining intact sites (m2 and m3) leading to preferential inhibition of branchpoint A3. Indeed, under conditions in which the m1 site was mutated, branchpoint A3 was preferentially inhibited as expected for a model involving flanking interaction motifs (Figure 4B, lanes 7,8). Consistent with this observation, the corresponding *in vitro* splicing gel showed that CUGBP2 inhibited the formation of one lariat intermediate but not the other (Figure S4, lanes m1). For the single mutant, m3, which should display the reciprocal pattern of inhibition by CUGBP2, branchpoints A1 and A2 were preferentially inhibited relative to A3 (Figure 4B, lanes 11,12). Finally, the triple mutant, m1,2,3, was tested. Here, the elimination of all three binding motifs neutralized the inhibitory effects of CUGBP2 on branchpoint formation (lanes 17,18). Again, these results were consistent with the splicing intermediates of these reactions (Figure S4, lanes m3 and m1,2,3). Adenovirus major late (Ad1) pre-mRNA was tested as a control, because Ad1 pre-mRNA lacks CUGBP2 motifs in the upstream intron. Both the *in vitro* splicing and branchpoint formation of Ad1 were unaffected by the addition of recombinant CUGBP2 (data not shown).

To determine which step before branchpoint formation is specifically affected by the addition of CUGBP2 to the splicing reaction, we analyzed complex assembly on the E5-10 RNA substrate in the presence or absence of recombinant CUGBP2. We demonstrate that CUGBP2 blocks U2 snRNP association because CUGBP2 inhibited complex A formation on the wild type substrate but not on the m1,2,3 mutant substrate (Figure S5A). The identity of the complex was verified as the U2 snRNP-containing complex A, since its assembly was inhibited by U2 snRNA cleavage (Figure S5A, S5B). In contrast, parallel samples assembled in the absence of ATP showed no effect of CUGBP2 on complex E assembly (Figure S5C).

The results shown above are consistent with a model in which site-specific binding of CUGBP2 surrounding the branch site region mediates exon skipping. Because exon definition could potentially affect branchpoint formation in the upstream intron by interactions involving U1 snRNP and U2AF across the exon, we asked whether strengthening the 5′ splice site of the NI exon would antagonize the silencing effect of CUGBP2. For this purpose, we increased the complementarity of the 5′ splice site to U1 snRNP and tested the ability of CUGBP2 to induce silencing *in vivo*. This mutation had no detectable effect on silencing by CUGBP2 (Figure S6). We also show that U2AF and CUGBP2 can contact the same RNA at the same time indicating that branchpoint inhibition occurs after U2AF but before U2 snRNP binding (Figure S7). This, together with the lack of effect of CUGBP2 on complex E, which contains U1 snRNP, U2AF, and SF1, is consistent with a mechanism involving inhibition at a step subsequent to exon definition. Thus, the inhibitory role of CUGBP2 is likely to involve direct antagonism of U2 snRNP binding at the NI branch site region.

### Autoregulation of CUGBP2 at the level of alternative splicing was revealed by a search for additional skipped exons with a similar arrangement of core and flanking motifs

A recent publication by Yeo, et al. (2007) used computational approaches to identify intronic splicing regulatory elements (ISREs) in the introns upstream and downstream from skipped exons [22]. One of the ISREs identified was a UGUGUU motif with the propensity to be found within 400 nucleotides of conserved skipped exons. The Yeo study identified 168 skipped exons with a UGUGUU motif in their upstream intron. We obtained this list for further analysis.

In order to extend our analysis to identify additional exons that are potentially silenced by CUGBP2, we searched the list of 168 exons for the following sequence features: (1) the presence of conserved pairs of UGUGU and GU motifs within 100 nucleotides of the 3′ splice site of the skipped exon with a spacing of 10–30 nucleotides between the motifs, and (2) the presence of potential branch site(s) between the motifs. Because the m1 and m2 motifs were sufficient to inhibit branchpoints A1 and A2 on the DUPNIwt substrate, we rationalized that two motifs flanking the branch site would be sufficient for the prediction of CUGBP2 regulation. Potential branch sites were required to match the human consensus sequence, YUNAY (Y, pyrimidine; N, any nucleotide) with one mismatch allowed [23]. From this analysis, we determined that 48 of the 168 exons (29%) fit these criteria. We chose 27 exons to test for regulation by CUGBP2.

**Figure 5:**
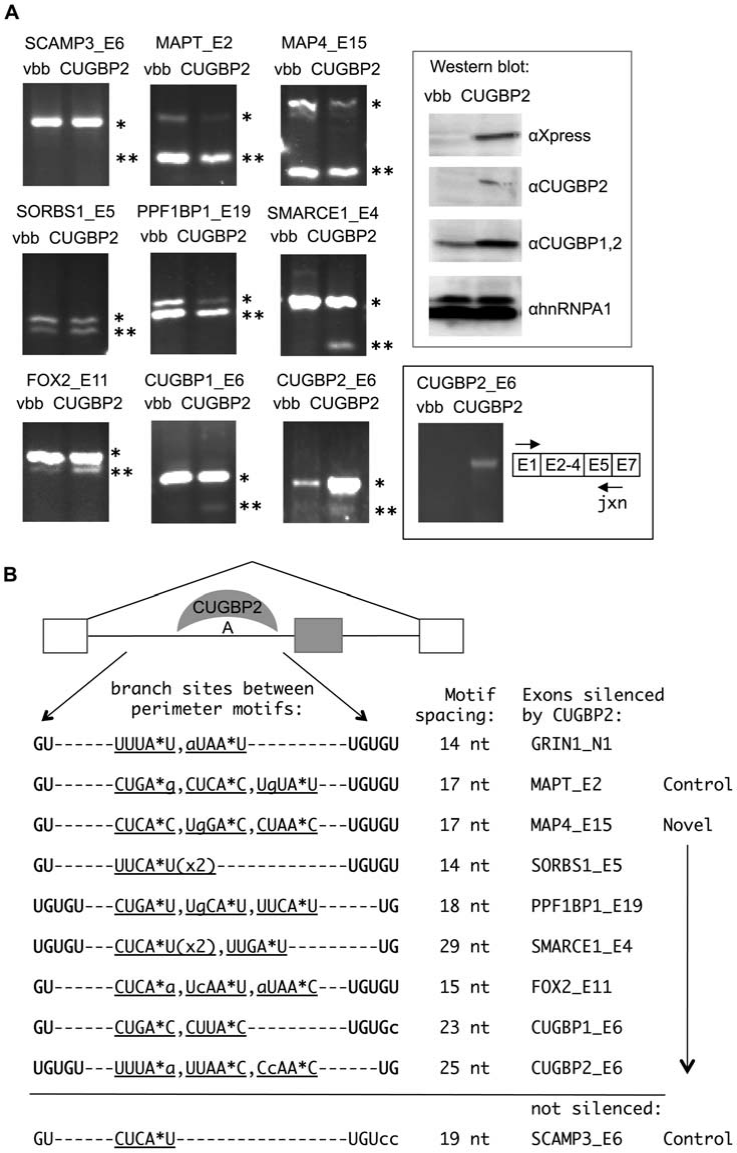
A motif code for splicing silencing reveals novel endogenous exons that are silenced by CUGBP2. (A) HEK293T cells were transfected for 36 hours with CUGBP2 protein expression vector (CUGBP2) or vector backbone control (vbb). Exon-included (*) and skipped (**) RT-PCR products are shown after separation on 2% agarose gels. The gene name and exon number are labeled above each panel. The ΔEI values are as follows: SCAMP3_E6, 0±0; MAPT_E2, −5.3±0.6; MAP4_E15, −11.7±3.5; SORBS1_E5, −8±1.7; PPF1BP1_E19, −12.3±2.1; SMARCE1_E4, −21.7±5.5; FOX2_E11, −10±2; CUGBP1_E6, −4.7±0.6; CUGBP2_E6, not determined; ±, standard deviation of three separate experiments. Inset: Western blot confirming CUGBP2 expression (αXpress); total CUGBP2 as detected by 1H2 antibody (αCUGBP2), CUGBP1 and 2 using 3B1 antibody (αCUGBP1,2), and loading control (αhnRNPA1). Black box: RT-PCR detection of CUGBP2 exon 6 skipped mRNA using primers specific for exon 1 and the exon 5/7 junction (jxn). (B) Model depicting the inhibition of branchpoint formation by the binding of CUGBP2 to flanking interaction sites. Below schematic: for exons silenced by CUGBP2, the predicted branch sites between the motifs are shown. Branchpoint adenosine (A*); lowercase letters indicate mismatches to the branch site consensus. Spacing in nucleotides (nt) between the perimeter motifs is shown for all tested exons.

To analyze the response of these endogenous exons to CUGBP2 overexpression, we optimized a calcium phosphate transfection method to obtain >90% transfection efficiency in HEK293T cells. HEK293T cells were chosen for these experiments, since there is no detectable expression of CUGBP2 (Figure 5A, Western blot). RNA was harvested from the cells and the test exon region was amplified by RT-PCR with primers specific for the flanking exons. Ten predicted exons showed an increase in exon skipping when CUGBP2 was overexpressed (Figure 5A, panels MAP4_E15, SORBS1_E5, PPF1BP1_E19, SMARCE1_E4, FOX2_E11, and CUGBP2_E6; not shown: NFAT_E2, CTBP1_E2, PTER_E3, and MLLT10_E13). Of the 17 exons that were not affected by CUGBP2, one was constitutively included and resistant to CUGBP2, 8 were not expressed in HEK293T cells, and 8 were always skipped, therefore, CUGBP2 could not induce additional skipping. Therefore, 10 out of 11 testable exons were regulated by CUGBP2 indicating that we have identified a specific code that can be used to accurately predict CUGBP2 regulation. For all of the confirmed target exons, the core and flanking motifs were separated by 14–29 nucleotides, and multiple branch site candidates were located between the CUGBP2 motifs (Figure 5B). Note that exon 6 of the SCAMP3 transcript, which contains two mismatches to the GU-rich motif pattern, was insensitive to silencing by CUGBP2. MAPT_E2 is shown as a positive control, because GRIN1 is not expressed in HEK293T cells [24].

An interesting observation was the appearance of an exon skipped product of the CUGBP2 transcript itself, which was specific for conditions in which CUGBP2 was overexpressed. However, the primers in this case also amplified mRNA expressed from transfected CUGBP2, thereby complicating interpretation. For this reason we designed a downstream primer specific for the junction between exons 5 and 7, since such a junction primer should amplify only the skipped product from the endogenous mRNA. The junction primer was used together with a forward primer specific for the first exon. The results with the junction primer clearly showed the accumulation of the exon 6 skipped version of the endogenous CUGBP2 transcript upon overexpression of CUGBP2 (Figure 5A, black box). Note that this primer did not amplify the CUGBP2 protein expression plasmid or mRNA from transfected CUGBP2 (data not shown). It is also important to note that although CUGBP2 protein is not detected by Western blotting, there are low levels of CUGBP2 RNA in HEK293T cells. This may indicate that trace amounts of the protein are present in these cells or that the mRNA is translationally repressed. Furthermore, because there is an enrichment of CUGBP2 protein in the rat cerebral cortex and a deficiency in the cerebellum [17], we predicted and confirmed that there would be more skipping of exon 6 in the cortex (data not shown). To establish the identity of the exon 6 skipped product, we cut the band out of the gel, cloned and sequenced it. The cloned product exactly matched the exon 5–7 junction sequence demonstrating its identity as the skipped product (data not shown).

We also tested an exon in the CUGBP1 transcript that is homologous to CUGBP2 exon 6 (CUGBP1_E6). Here, there is one mismatch to the core motif and although CUGBP2 can silence this exon, the effect is less than other target exons with perfect matches to the consensus motifs. This suggests that CUGBP2 regulation can be titrated depending on the sequence content and binding affinity to target motifs.

### CUGBP2 silences its own exon through interaction sites flanking the branch site region

**Figure 6:**
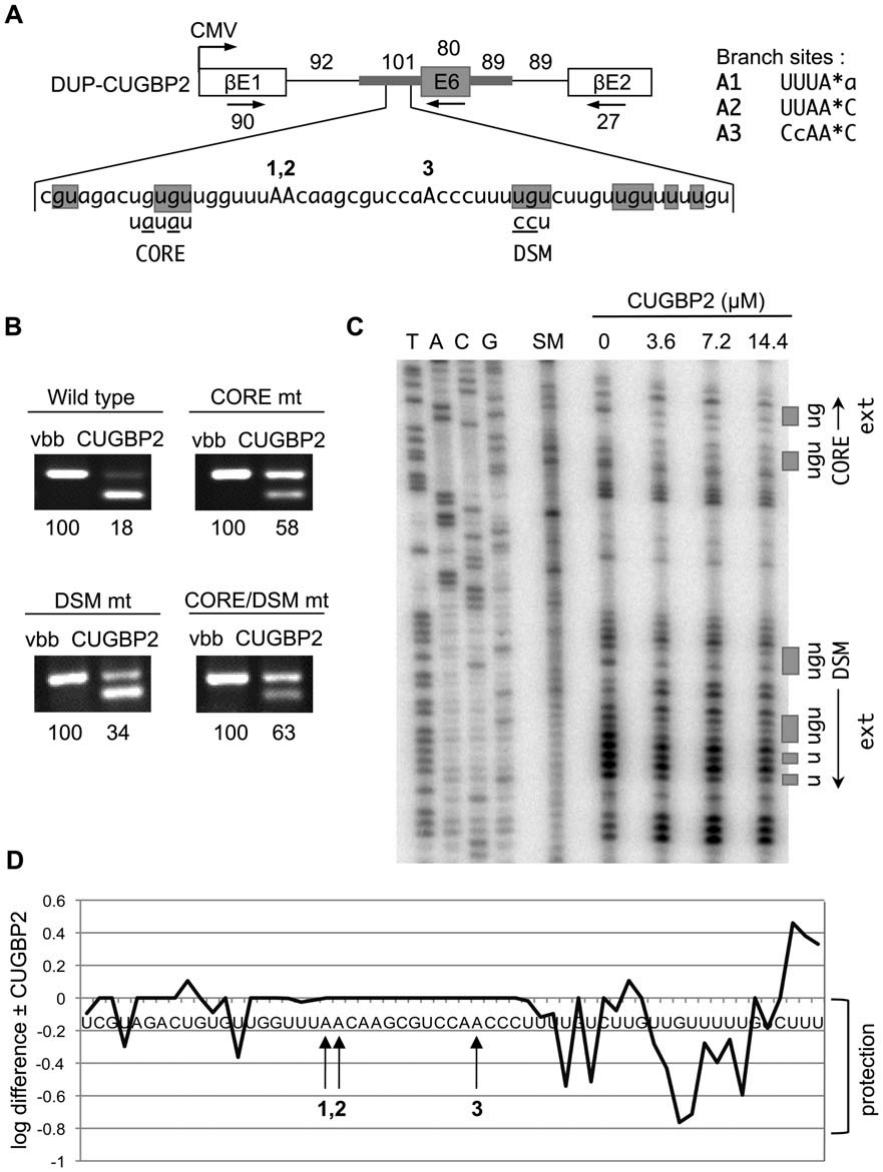
CUGBP2 is autoregulated by silencing from exon 6 branch site perimeters. (A) Splicing reporter with sequence showing predicted branch sites (A1, A2, A3) and CUGBP2 binding motifs (shaded regions). Primers (arrows) used for RT-PCR (βE1 and βE2) or footprinting analysis (E6) are shown. Mutations in core (CORE) and downstream (DSM) motifs are indicated. (B) Autoregulation of E6 depends upon CUGBP2 binding motifs surrounding the branch site region. Gel panels represent RT-PCR analysis of the wild type or mutant derivatives of the splicing reporter co-expressed with vector backbone control (lanes vbb) or CUGBP2 protein expression vector (lanes CUGBP2) in HEK293T cells. Percent exon inclusion values are shown below gel panels. (C) Footprinting analysis of the predicted branch site region upstream of E6 of CUGBP2 pre-mRNA. Starting material (lane SM), or RNA treated with CMCT in the absence (lane 0) or presence (lanes 3.6, 7.2, 14.4 µM) of CUGBP2 protein. Protected regions are indicated as shaded boxes at right with extended regions of protection (ext) from the CORE or DSM motifs indicated by arrows. (D) Log difference of the band intensities of modified nucleotides in the absence or presence of 14.4 µM CUGBP2. Negative values represent regions protected by CUGBP2. Note that the strength of protection decreases with increasing distance from the primer used for primer extension.

To determine whether CUGBP2 silences its own exon by a mechanism similar to that shown for the NI exon, exon 6 and the adjacent introns of CUGBP2 pre-mRNA were cloned into the DUP splicing reporter (Figure 6A). In this context, overexpression of CUGBP2 had a robust silencing effect changing the exon 6 splicing pattern from 100% to 18% inclusion in transfected HEK293T cells (Figure 6B, lanes Wild type). To address the functional significance of the CUGBP2 binding sites at the boundaries of the predicted branch sites, we tested site-directed mutations in the core (CORE) and downstream (DSM) motifs (Figure 6A). One perfect match to the branch site consensus (A2) and two additional candidates with a single mismatch (A1,A3) are the only plausible branch sites located within 100 nucleotides of the 3′ splice site of exon 6. Mutations in the CORE and DSM motifs resulted in a reduction of splicing silencing by CUGBP2 (Figure 6B, lanes CORE mt, DSM mt), and these effects were additive in the double mutant (lanes CORE/DSM mt). These are similar to the results shown for the NI exon, providing additional support for the perimeter-binding model. We note that although mutations did not completely eliminate CUGBP2 regulation of exon 6, footprinting experiments documented additional contact points extending from the CORE and DSM motifs suggesting that alternate binding registers might allow for some residual silencing (see below). Also note that we tested the possible role of an intronic UGUGU motif located 70 nucleotides downstream from exon 6. Mutation of this motif to UAUAU had a negligible effect on splicing silencing by CUGBP2, ruling out effects across the exon and further supporting our model (data not shown). Furthermore, we show that CUGBP1 is also a weak silencer of this exon, but does not act through the CORE and DSM motifs like CUGBP2 (Figure S3A, lanes CUGBP2_E6).

Finally, we used RNA footprinting to identify CUGBP2 contact sites in the neighborhood of the predicted branch sites upstream of exon 6 (Figure 6C and 6D). GU-rich motifs flanking A1, A2, and A3 were protected by the addition of purified CUGBP2 similar to that observed above for the NI 3′ splice site (Figure 6C, last three lanes at right). That is, two protected regions at the borders of the predicted branch sites overlap with the UGUGU core and UG flanking motifs in agreement with the perimeter-binding model. A difference in the pattern of protection, however, was the finding that two sets of motifs on either side of the branch site region extend outward, indicating variations in the mode of binding compared to the NI exon.

## Discussion

### A branch site-perimeter–binding model for alternative exon silencing

In this study we focused on the silencing face of the dual functional splicing factor, CUGBP2, to understand how it recognizes and silences the NI cassette exon of the NMDA R1 receptor. The first hint of how this exon target is recognized was revealed by chemical modification RNA footprinting of a high affinity binding region, which showed two contact sites—a core UGUGU and flanking GU—closely positioned in the neighborhood of the predicted branch sites. These contact sites and a third weaker footprinting site were shown to modulate alternative splicing of the NI exon *in vivo*. Furthermore, the positions of the branch sites were mapped between the core and flanking motifs. These branch sites were collectively inhibited by CUGBP2 with a dependence on the presence of flanking GU-rich binding motifs. Thus, guilt-by-association places CUGBP2 at the boundaries of the branch sites it regulates in support of the three-motif occupancy model illustrated in Figure 4A. The regulation of an ensemble of branchpoints by a perimeter-type binding model, and the discovery that an exon in the CUGBP2 transcript is itself silenced by a similar arrangement of binding motifs, are novel findings of this study.

We show additional support for this model by identifying novel skipped exons that are functionally silenced by CUGBP2 based on database searches for similarities to the configuration of NI regulatory motifs. These confirmed targets contained the characteristic pattern of candidate branch sites flanked by GU and UGUGU motifs, which were themselves separated by ∼20 nucleotides in the adjacent 3′ splice site region (Figure 5). Notably, exon 6 of the CUGBP2 transcript was the most interesting member of this group due to the implications for autoregulation. Thus, the specific arrangement of CUGBP2 binding motifs around the branch sites of the NI exon is a silencing code that can be generalized to have a functional impact on other skipped exons.

Our results support and extend those of a previous study, which reported the identification of a UGUGUU motif as an intronic splicing regulatory element (U17) enriched within 400 nucleotides upstream of conserved skipped exons [22]. This previous study reported the association of the U17 element with exon inclusion in brain tissue as indicated by microarray analysis. In contrast, our study shows that the UGUGU core of the U17 element is generally associated with exon silencing when the motif is paired with a flanking GU surrounding the functional branch sites. This is not necessarily a discrepancy, but more likely a reflection of a mechanism operating on a subset of exons containing a U17-related element. Here we demonstrate the value of the branch site as a functional reference point that can be used together with the precise binding interaction motifs of a splicing factor to computationally predict new splicing regulatory targets. Our results are consistent with the types of binding motifs identified for ETR-3 using a SELEX approach, although the relationship of the binding motifs to the branch site region and the autoregulatory role of CUGBP2 were not determined [13]. Furthermore, the types of motifs identified for Bruno-like proteins in the α-actinin transcript are in agreement with our results [25].

Branchpoint formation reflects a critical step in the catalysis of the splicing reaction, but its role in the regulation of alternative splicing across the transcriptome represents largely uncharted territory. Only a small number of branchpoints have been experimentally mapped, however, and there are often multiple candidate branch sites in the 3′ splice site region that match the consensus sequence [23]. Examples of alternative splicing regulation through the use of a suboptimal branchpoint include the calcitonin/calcitonin gene-related peptide exon 4 and fibroblast growth factor receptor 2 exon IIIc [26]–[29]. Branch site selection has also been implicated in the regulation of mutually exclusive exons of beta tropomyosin and in the processing of human growth hormone pre-mRNA [30],[31].

What advantages would a perimeter-binding model provide for the control of access to the branch site region? The pre-mRNA branch site and flanking sequences are sequentially contacted by several factors during spliceosome assembly [32],[33]. The branch site interacts with the RS domain of U2AF^65^ bound to the polypyrimidine tract of the 3′ splice site, followed by interactions with the RS domain of an SR splicing factor bound to an enhancer element in the adjacent exon [34]–[36]. Splicing Factor 1 (SF1) makes direct contacts with the branch site during complex E assembly [37],[38]. In complex A, SAP155 binds to sites flanking the branch site and replaces SF1 to recruit U2 snRNP [39],[40]. Here multiple contact sites may furnish CUGBP2 with the added stability to inhibit the association of U2 snRNP with the branch site region and/or may block conformational transitions of the spliceosome [41]. The perimeter-type binding model described here is significant in allowing for the coordinate regulation of multiple branchpoints to control alternative splicing of a cassette exon. Moreover, the distinctive pattern of RNA motif recognition by CUGBP2 may facilitate its enhancing roles in other contexts.

CUGBP2 is a modular protein containing three RNA recognition motifs (RRMs) in which a divergent domain of unknown function separates RRMs 2–3. The domain structure of the protein may be geared to facilitate binding of a monomer to a pair of core and flanking motifs forming a bridge between them as our model indicates. Alternatively, a single monomer might bind to all three GU-rich motifs. Both models would limit access to the branch sites by factors sliding along the RNA from upstream and downstream directions. Our footprinting results with CUGBP2 are in agreement with previous structural studies showing that a single RRM can contact ∼2–7 nucleotides of its bound RNA ligand, but additional studies will be required to understand the topology of binding associated with its silencing function [42]–[44]. Given the inherent flexibility of RNA binding proteins, it would not be surprising that breathing motions of CUGBP2 could adjust the relative conformations of the RRM domains to optimize recognition specificity in different sequence contexts.

Autoregulation has been shown for a growing number of splicing factors, including PTB, FOX-2, Nova-1, SRp20, SC-35, TIA1, and TIAR [45]–[48]. Here we dissect the mechanism of CUGBP2 autoregulation. CUGBP2 acts functionally through motifs surrounding the branch site region to silence exon 6 near the 5′ end of its own transcript. Conceptual translation reveals that skipping of this exon causes a shift in the reading frame, which introduces a premature termination codon in the exon 7 region of the transcript. In this way, the resulting transcript could be targeted for nonsense-mediated mRNA decay. Conversely, translation of the exon 6 skipped transcript could generate a truncated protein ending within RRM2. The advantage of a tight motif arrangement around the branch site region would be to dynamically adjust exon 6 inclusion based on fluctuations in the levels of CUGBP2 protein.

The observation that CUGBP2 can cross regulate exons in the CUGBP1 and FOX2 transcripts, and that CUGBP1 can silence CUGBP2 exon 6 to a lesser extent, implicates CUGBP2 in a network of splicing factor regulation (Figure 5 and Figure S3). We speculate that this may be important in specifying neural cell identity and for fine-tuning of neural exon splicing. In the future, it would be of interest to study the differences in binding specificities and target exon selection by CUGBP1 and CUGBP2.

The binding of CUGBP2 to the perimeters of the branch sites allows for sensitive gradations specifying the levels of NI exon inclusion. Because the peptide region encoded by the NI exon modulates sensitivity of the NMDA receptor to zinc ions, protons, and polyamines, such a mechanism would be advantageous for fine-tuning this modular property of receptor function in different regions of the brain or during development [49]. The biochemical functions of NMDA receptors are of fundamental importance in synaptic plasticity where deficits in this subunit are associated with altered brain function in the context of Alzheimer's disease. The CI cassette exon, which is regulated by the enhancing face of CUGBP2, encodes a functionally important region of the receptor involved in membrane trafficking and signaling to the nucleus. Our study provides the starting point to investigate the broader roles of CUGBP2 in regulation of the CI cassette and additional exons throughout the transcriptome. Insights from this study can also be applied to systematically examine the role of intronic mutations in the neighborhood of the branch site underlying mechanisms of human disease.

## Methods

### Plasmid construction

The mouse CUGBP2 (pcDNA4/NAPOR) and rat PTB (pcDNA4/PTB) protein expression vectors were described previously [17]. The Nova-1 protein expression vector was a gift of Robert Darnell [50] and the CUGBP1 expression vector was a gift of Thomas Cooper [51]. To generate the DUPNIwt and DUP-CUGBP2 splicing reporters, the cassette exon and flanking introns were amplified from rat genomic DNA and inserted between the ApaI and BglII restriction sites of the DUP4-1 splicing reporter [52]. The DIPm93wt splicing reporter [53] was used to generate DIPNIwt and mutant derivatives. DIPNIwt was generated by insertion of a 39 base pair fragment containing the 3′ splice site region of the NI exon at position −13 base pairs upstream from the test exon. pBSDUPNI wild type and mutant vectors for *in vitro* transcription were generated by PCR amplification from the DUPNIwt or mutant splicing reporters and insertion between the HindIII and EcoRI restriction sites of the pBS- phagemid vector (Stratagene). E5-8, E5-10, and E5-15 plasmids were generated in a similar manner. Plasmids were confirmed by restriction digestion and DNA sequencing.

### Nitrocellulose filter binding

RNA substrates were ^32^P-UTP-labeled by *in vitro* transcription and used at a final concentration of 1000 cpm/µl (20–100 nM). RNA was heated at 85°C for 5 min and then cooled to 37°C for 5 min to remove long-range secondary structures. Serial dilutions of CUGBP2 protein were prepared on ice in binding buffer (50 mM Tris pH 8.0, 150 mM NaCl, 0.1 mg/ml tRNA, 2 mM DTT, 20 units RNasin (Promega)) in a final volume of 199 µl. Protein samples were warmed to 37°C for 5 min before adding 1 µl of diluted RNA (final RNA concentration 100–500 pM). RNA-protein complexes were assembled in triplicate at 37°C for 30 min before filtration through 25 mm BA85 filters backed by DE81 filters in a Millipore 1225 vacuum manifold. Filters were separated and dried at room temperature overnight. The cpm retained on the BA85 filter corresponded to RNA bound to protein and cpms retained on the DE81 filter corresponded to free RNA. Bound/total RNA was plotted as a function of increasing protein concentration using KaleidaGraph Synergy Software and data were fit to a hyperbola to estimate the dissociation constant (K_d_) according to the equation bound/total RNA = [CUGBP2]/([CUGBP2]+K_d_).

### Chemical modification footprinting

RNA-protein complexes were assembled for 30 min at 37°C with 0.18 µM RNA substrate and purified recombinant CUGBP2 protein in a final volume of 50 µl. Each sample was then combined with an equal volume of 42 mg/ml 1-cyclohexyl-3-(2-morpholinoethyl)-carbodiimidemetho-*p*-toluene-sulfonate (CMCT) and chemical modification was carried out at 37°C for 7 minutes. Reactions were terminated by ethanol precipitation. Recovered RNA was treated with proteinase K followed by phenol chloroform extraction and ethanol precipitation. Primer extension was carried out with a 5′^32^P-labeled primer using Superscript II reverse transcriptase (Invitrogen). Sequencing ladders were generated using Thermo Sequenase Cycle Sequencing Kit (USB) according to manufacturers protocol. cDNA was separated on a 10% polyacrylamide gel and gel images were recorded on a BAS-2500 Phosphorimager (Fujifilm).

### Splicing reporter assay

C2C12 and N18TG2 cells were grown in DMEM, 10% (v/v) fetal bovine serum (FBS). Twenty-four hours prior to transfection 1.5×10^5^ C2C12 cells or 2×10^5^ N18TG2 cells were seeded on 35-mm plates to achieve 60–80% confluency. For transient transfection, 1 µg pcDNA4 His/Max vector backbone or 1 µg pcDNA4/CUGBP2, pC1-Nova-1, pcDNA4/PTB, or pcDNA3.1/CUGBP1 expression vector and 0.25 µg splicing reporter were mixed with 250 µl Opti-MEM followed by addition of an equal volume of Opti-MEM mixed with 2.5 µl Lipofectamine 2000 (Invitrogen) and incubated at room temperature for 20 min. Media on cells was replaced with 1.5 ml DMEM, 10% (v/v) FBS prior to transfection. Total RNA was isolated 36 hours after transfection using TRIZOL reagent (Invitrogen). Two µg total RNA was reverse transcribed as described previously [17]. PCR was carried out in 10 µl reactions containing 1 µl of the reverse transcription reaction, 0.1 µM of each primer, 2 mM MgCl_2_, 0.2 mM dNTPs, and 2.5 units Taq DNA polymerase (Promega). Forward primers were 5′^32^P-labeled. Cycling parameters were adjusted to give amplification in the linear range. Conditions were as follows: denaturation 94°C, 1 min, annealing at 60°C, 1 min, and elongation at 72°C, 1 min for 22 cycles followed by a final elongation step at 72°C for 10 min. PCR samples were resolved on 6% polyacrylamide/5 M urea gels. Data were quantified using a BAS-2500 Phosphorimager system and Image Gauge software.

### *In vitro* splicing and branchpoint analysis

pBSDUPNIwt or mutant derivatives were digested with EcoRI for *in vitro* transcription in the presence of α^32^P-UTP. *In vitro* splicing assays were carried out for 45 min or 1 hour as previously described [54] except MgCl_2_ was at a final concentration of 2.2 mM. Branchpoints were detected by primer extension from parallel splicing reactions constituted with unlabeled pre-mRNA in a reaction containing 200 U MMLV reverse transcriptase (Invitrogen), 10 mM DTT, 1 mM dNTPs, 1× first strand buffer, and 50 nM 5′^32^P-labeled primer in a total reaction volume of 20 µl. Reactions were incubated at 37°C for 30 minutes and were terminated by ethanol precipitation. Samples were resuspended in 6 µl formamide loading buffer and 1/3 of the sample was separated on an 8% polyacrylamide/7 M urea gel next to a sequencing ladder. Gel images were recorded on a BAS-2500 Phosphoimager system.

### Spliceosome assembly

Spliceosome complexes were assembled on the E5-10 wild type or mutant RNA in a 10 µl reaction containing 20 mM Hepes, pH 7.4, 44% Hela nuclear extract, 2.2 mM MgCl_2_, 60 mM KCl, 1.5 mM ATP, 5 mM creatine phosphate, and 0, 1.6, or 3.2 µM CUGBP2 at 30°C for 15 minutes. Spliceosome assembly was stopped by the addition of heparin at a final concentration of 2 mg/ml and incubation for an additional 3 minutes. Half of the reaction was separated on a 3.75% native polyacrylamide gel cast in 50 mM tris-glycine buffer and run at 4 watts at 4°C for 4 hours. Gels were dried under vacuum and visualized by phosphoimager. For assembly of the ATP-independent E complex, the nuclear extracts were preincubated at 30°C for 10 minutes to deplete ATP and complexes were assembled in the absence of ATP or creatine phosphate for 8 minutes. Oligonucleotide-directed cleavage of U1 and U2 snRNAs was carried out as described previously [55].

### Calcium phosphate transfection for analysis of endogenous mRNA targets of CUGBP2

HEK293T cells were grown in DMEM, 10% (v/v) FBS. Twenty-four hours prior to transfection, 2×10^5^ HEK293T cells were seeded in 35-mm dishes precoated with poly-L-lysine (Sigma). Cells were approximately 50% confluent at the time of transfection. Before transfection, all reagents were brought to room temperature. For one well, 1.6 µl of 1 µg/µl CUGBP2 protein expression vector or vector backbone was mixed with 16.1 µl 2.5 M CaCl_2_ by vortexing briefly. Next, 65.8 µl water was added and the CaCl_2_-DNA mixture was pipetted over 83.5 µl 2× BBS, pH 7.15 (50 mM N,N-bis(2-hydroxyethyl)-2-aminoethane sulfonic acid, 280 mM NaCl, 1.5 mM Na_2_HPO_4_) and vortexed for 3 seconds. Mixtures were incubated at room temperature for 10 minutes before 164 µl was added drop wise to each dish. After transfection, cells were incubated at 3% CO_2_ for 36 hours prior to RNA isolation using TRIZOL reagent (Invitrogen). RT-PCR was carried out as described above with unlabeled primers (Table S1). PCR samples were resolved on 2% agarose gels and quantified using Image Gauge software.

## Supporting Information

Figure S1 PTB and U2AF^65^ bind to the polypyrimidine tract between GU-rich motifs (shaded regions). PTB can effectively compete with CUGBP2 for binding to the core and upstream motifs. Gel: CMCT modification footprint with recombinant PTB and purified Hela U2AF. Left lane of each set, no protein; gray wedge, 1.8, 3.6, and 7.2 µM protein added. Right panel: Competition footprint; left lane, no protein; right lanes, 3.6 µM CUGBP2; black wedge, 0, 3.6, and 7.2 µM PTB added. PTB footprint (UCUUCUUCU) is underlined, U2AF footprint (CUUCUU) is boxed in schematic.
(1.85 MB TIF)

Figure S2 Quantitative Western blot of whole cell lysates demonstrates that endogenous CUGBP2 levels are low in C2C12 and N18TG2 cells. Whole cell lysates (WCL) were obtained from 90% confluent 10-cm dishes of each cell type as indicated. Western blot was carried out with antibodies specific for CUGBP2 (1H2), CUGBP1/2 (3B1), or hnRNPA1 (9H10) as indicated at right. Left panel is Western blot of recombinant CUGBP2 as a control for antibody sensitivity and relative protein levels.
(0.17 MB TIF)

Figure S3 CUGBP2 regulatory motifs are specific. (A) Splicing reporters were transfected into C2C12 cells in the presence or absence of recombinant CUGBP1 as indicated (top) and included and skipped forms of spliced reporter RNA were assayed by RT-PCR and separated on a polyacrylamide gel. Percent exon inclusion, EI (%), and change in percent exon inclusion with CUGBP1 overexpression are indicated below gel panels. The effect of CUGBP2 overexpression is shown for comparison (CUGBP2). (B) Splicing reporter assays were carried out as in (A) except Nova and PTB protein expression vectors were used.
(0.76 MB TIF)

Figure S4 Recombinant CUGBP2 inhibits splicing of the intron upstream of the NI cassette exon in vitro. In vitro splicing reactions were carried out using the two exon reporters DUPNIwt and mutant derivatives m1, m3, and m1,2,3. The presence (+) or absence (−) of ATP or recombinant CUGBP2 (CUGBP2) and the time of incubation are indicated at top of the gel. The structures of RNA intermediates and products are indicated at right. Note that the time dependence of the accumulation of branchpoints mapped in Figure 4 coincides with the appearance of the intron lariat-3′ exon intermediate in the in vitro splicing reactions shown above. The doublet band in the vicinity of the intron lariat is consistent with branchpoints at varying distances from the 3′ splice site (see for example, lane 6 from left).
(0.98 MB TIF)

Figure S5 Recombinant CUGBP2 inhibits U2 snRNP binding and complex A but not complex E formation. (A) CUGBP2 inhibits complex A formation and U2 snRNP binding. Splicing complex formation was carried out in the presence or absence of ATP, CUGBP2, or oligonucleotide-directed cleavage of U2 snRNA as indicated at top on either wild type (wt) or triple mutant (m1,2,3) E5-10 RNA substrates as indicated below. The position of the ATP independent complexes E and H are shown at left and the ATP-dependent complex A is shown at right. (B) Confirmation of oligonucleotide-directed cleavage of U1 snRNA and U2 snRNA (indicated at top). Positions of uncleaved U2 snRNA and U1 snRNA are shown at left. (C) CUGBP2 does not inhibit complex E formation. Splicing complex formation was carried out as in (A) except ATP was omitted from the reactions.
(1.27 MB TIF)

Figure S6 Strengthening the NI cassette exon 5′ splice site complementarity to U1 snRNP does not affect splicing silencing by CUGBP2. (A) Schematic of mutations made to the 5′ splice site to strengthen U1 snRNP binding (shaded nucleotides). (B) In vivo splicing assay with overexpression of a vector backbone control (vbb) or CUGBP2 protein expression vector (CUGBP2). Graph shows % exon inclusion values; error bars, standard deviations.
(0.15 MB TIF)

Figure S7 CUGBP2 and U2AF can bind to the same RNA at the same time. Increasing amounts of recombinant wild type or mutant CUGBP2 containing only RRMs 1 and 2 (RRM1_2) or RRMs 2 and 3 (RRM2_3) were bound to E5-10 RNA in the presence or absence of Hela purified U2AF. Protein concentrations are labeled on the top of the gel and free RNA and RNA-protein complexes are labeled at right.
(0.70 MB TIF)

Table S1 Primers used for analysis of endogenous target exons shown in Figure 5.
(0.03 MB XLS)
