# Supplementary figures and images for: The CUGBP2 Splicing Factor Regulates an Ensemble of Branchpoints from Perimeter Binding Sites with Implications for Autoregulation
Source: PLoS Genet. 2009 Aug 14;5(8):e1000595. doi: 10.1371/journal.pgen.1000595 (PMC2715136; doi:10.1371/journal.pgen.1000595)

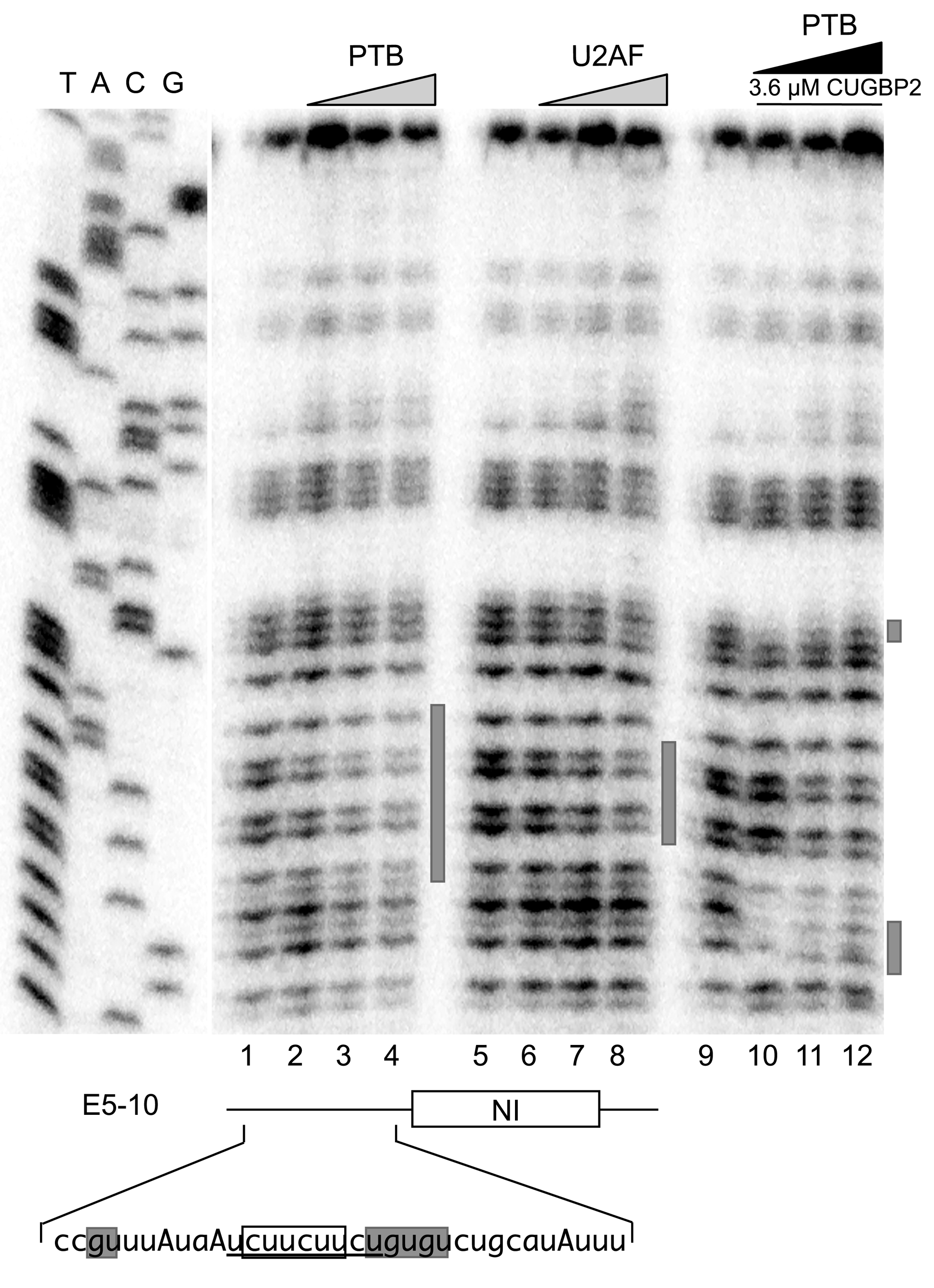

Supplement: Figure S1 — PTB and U2AF65 bind to the polypyrimidine tract between GU-rich motifs (shaded regions). PTB can effectively compete with CUGBP2 for binding to the core and upstream motifs. Gel: CMCT modification footprint with recombinant PTB and purified Hela U2AF. Left lane of each set, no protein; gray wedge, 1.8, 3.6, and 7.2 µM protein added. Right panel: Competition footprint; left lane, no protein; right lanes, 3.6 µM CUGBP2; black wedge, 0, 3.6, and 7.2 µM PTB added. PTB footprint (UCUUCUUCU) is underlined, U2AF footprint (CUUCUU) is boxed in schematic. (1.85 MB TIF) [file pgen.1000595.s001.tif]

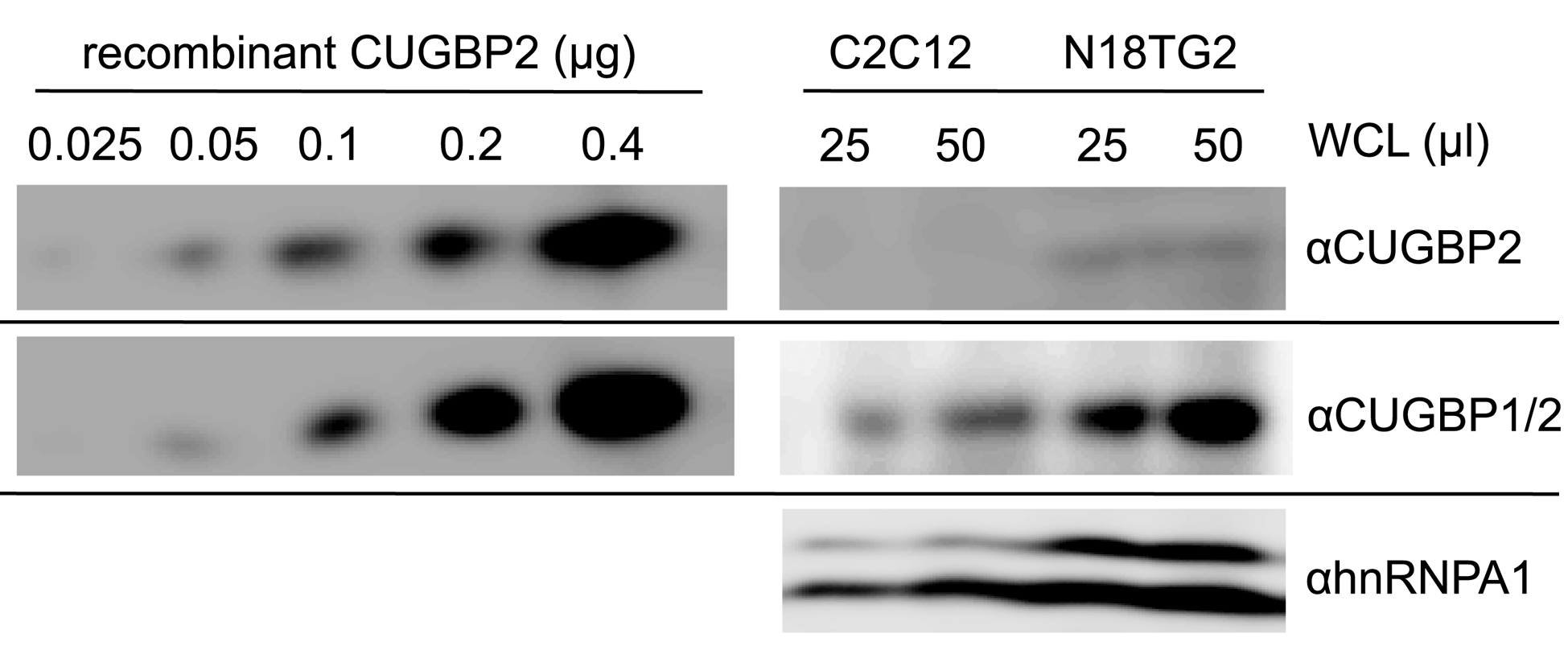

Supplement: Figure S2 — Quantitative Western blot of whole cell lysates demonstrates that endogenous CUGBP2 levels are low in C2C12 and N18TG2 cells. Whole cell lysates (WCL) were obtained from 90% confluent 10-cm dishes of each cell type as indicated. Western blot was carried out with antibodies specific for CUGBP2 (1H2), CUGBP1/2 (3B1), or hnRNPA1 (9H10) as indicated at right. Left panel is Western blot of recombinant CUGBP2 as a control for antibody sensitivity and relative protein levels. (0.17 MB TIF) [file pgen.1000595.s002.tif]

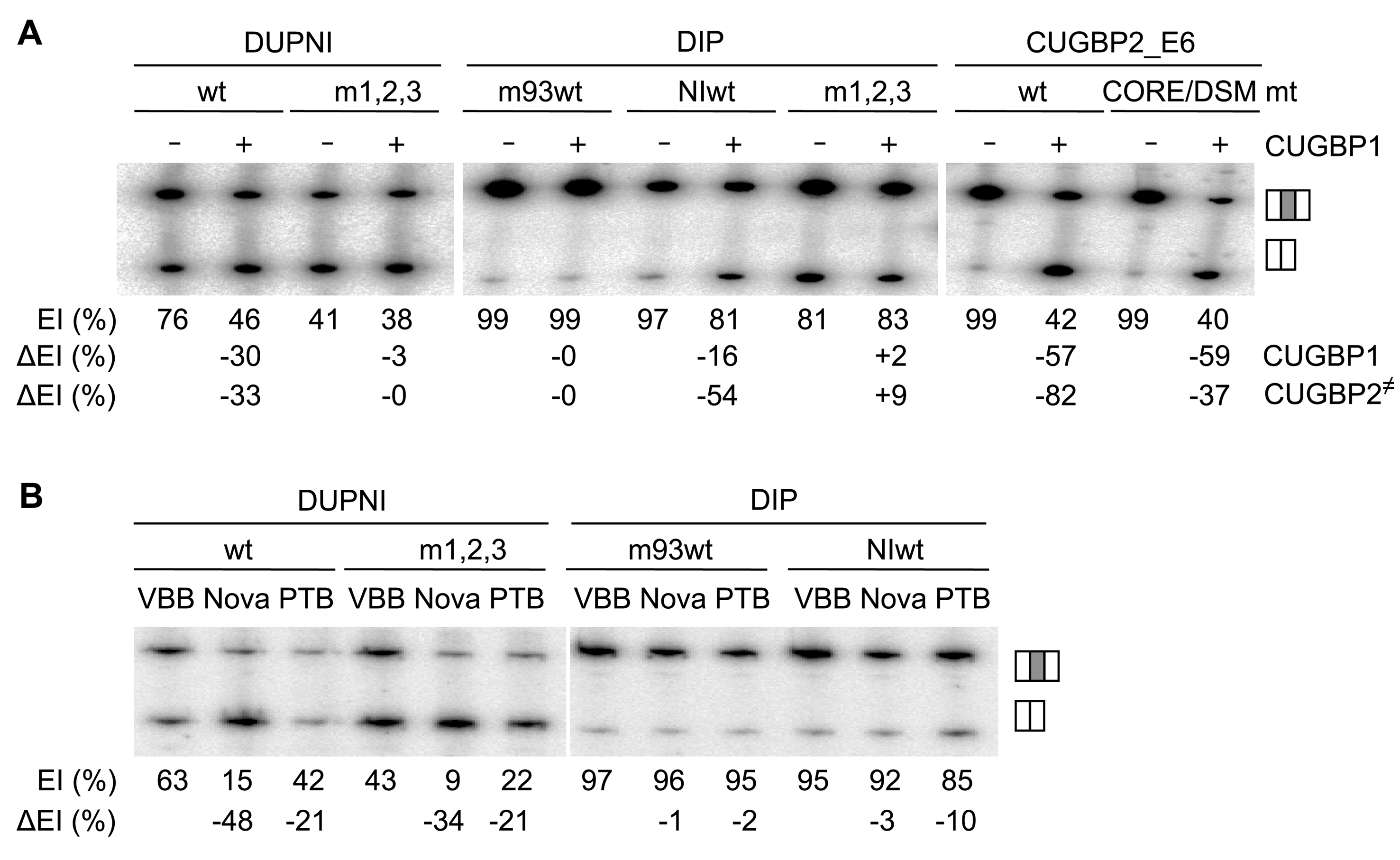

Supplement: Figure S3 — CUGBP2 regulatory motifs are specific. (A) Splicing reporters were transfected into C2C12 cells in the presence or absence of recombinant CUGBP1 as indicated (top) and included and skipped forms of spliced reporter RNA were assayed by RT-PCR and separated on a polyacrylamide gel. Percent exon inclusion, EI (%), and change in percent exon inclusion with CUGBP1 overexpression are indicated below gel panels. The effect of CUGBP2 overexpression is shown for comparison (CUGBP2). (B) Splicing reporter assays were carried out as in (A) except Nova and PTB protein expression vectors were used. (0.76 MB TIF) [file pgen.1000595.s003.tif]

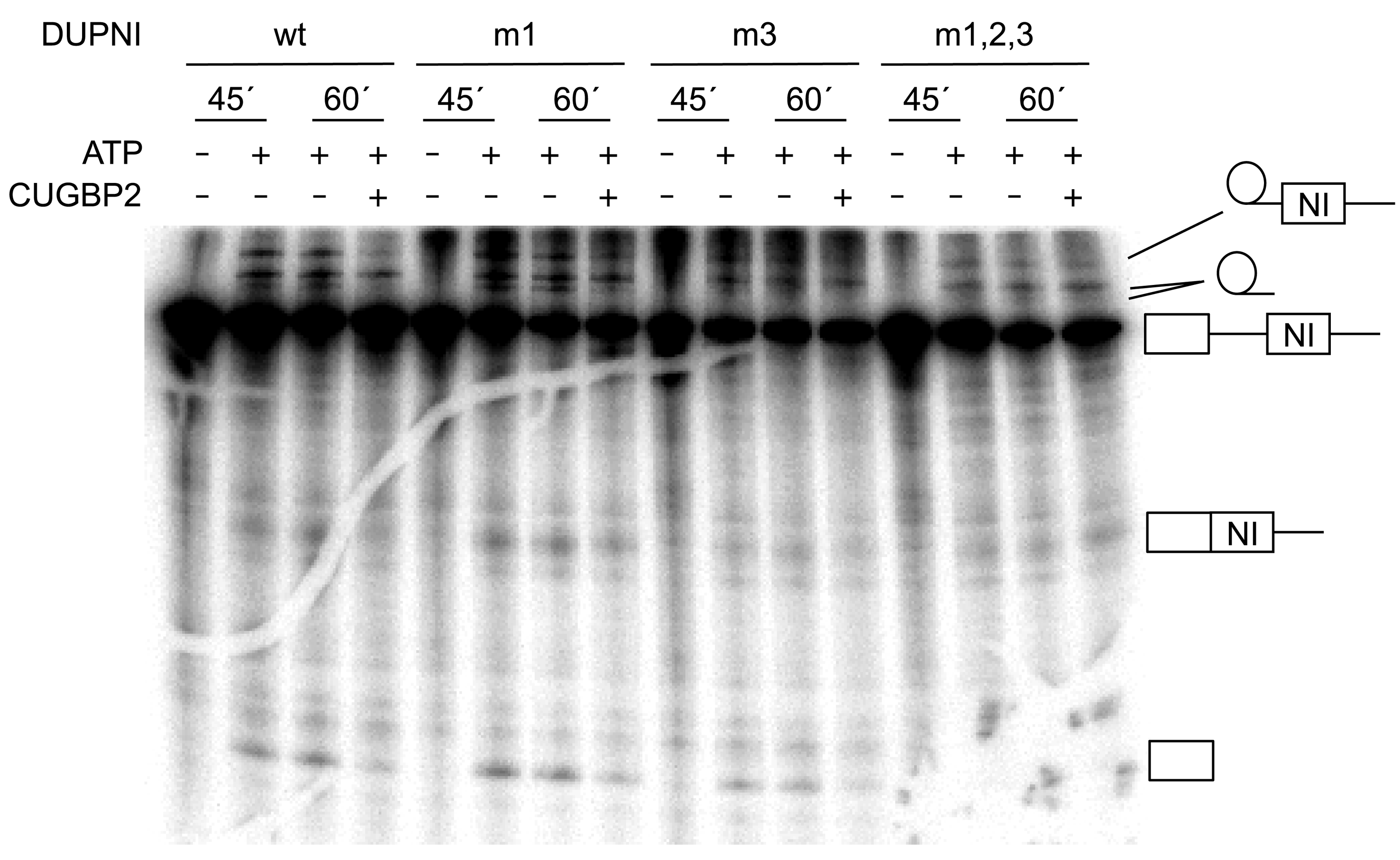

Supplement: Figure S4 — Recombinant CUGBP2 inhibits splicing of the intron upstream of the NI cassette exon in vitro. In vitro splicing reactions were carried out using the two exon reporters DUPNIwt and mutant derivatives m1, m3, and m1,2,3. The presence (+) or absence (−) of ATP or recombinant CUGBP2 (CUGBP2) and the time of incubation are indicated at top of the gel. The structures of RNA intermediates and products are indicated at right. Note that the time dependence of the accumulation of branchpoints mapped in Figure 4 coincides with the appearance of the intron lariat-3′ exon intermediate in the in vitro splicing reactions shown above. The doublet band in the vicinity of the intron lariat is consistent with branchpoints at varying distances from the 3′ splice site (see for example, lane 6 from left). (0.98 MB TIF) [file pgen.1000595.s004.tif]

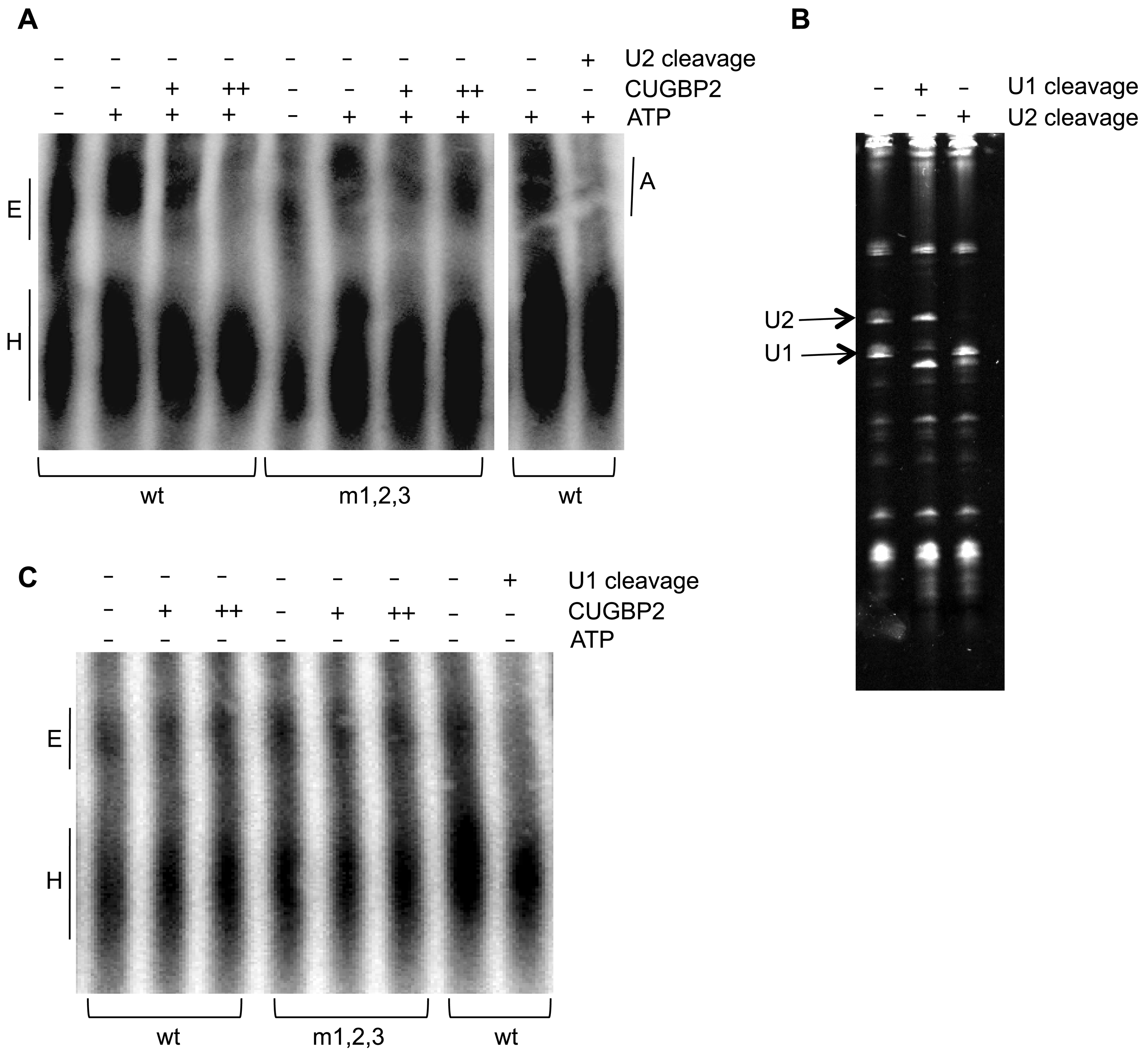

Supplement: Figure S5 — Recombinant CUGBP2 inhibits U2 snRNP binding and complex A but not complex E formation. (A) CUGBP2 inhibits complex A formation and U2 snRNP binding. Splicing complex formation was carried out in the presence or absence of ATP, CUGBP2, or oligonucleotide-directed cleavage of U2 snRNA as indicated at top on either wild type (wt) or triple mutant (m1,2,3) E5-10 RNA substrates as indicated below. The position of the ATP independent complexes E and H are shown at left and the ATP-dependent complex A is shown at right. (B) Confirmation of oligonucleotide-directed cleavage of U1 snRNA and U2 snRNA (indicated at top). Positions of uncleaved U2 snRNA and U1 snRNA are shown at left. (C) CUGBP2 does not inhibit complex E formation. Splicing complex formation was carried out as in (A) except ATP was omitted from the reactions. (1.27 MB TIF) [file pgen.1000595.s005.tif]

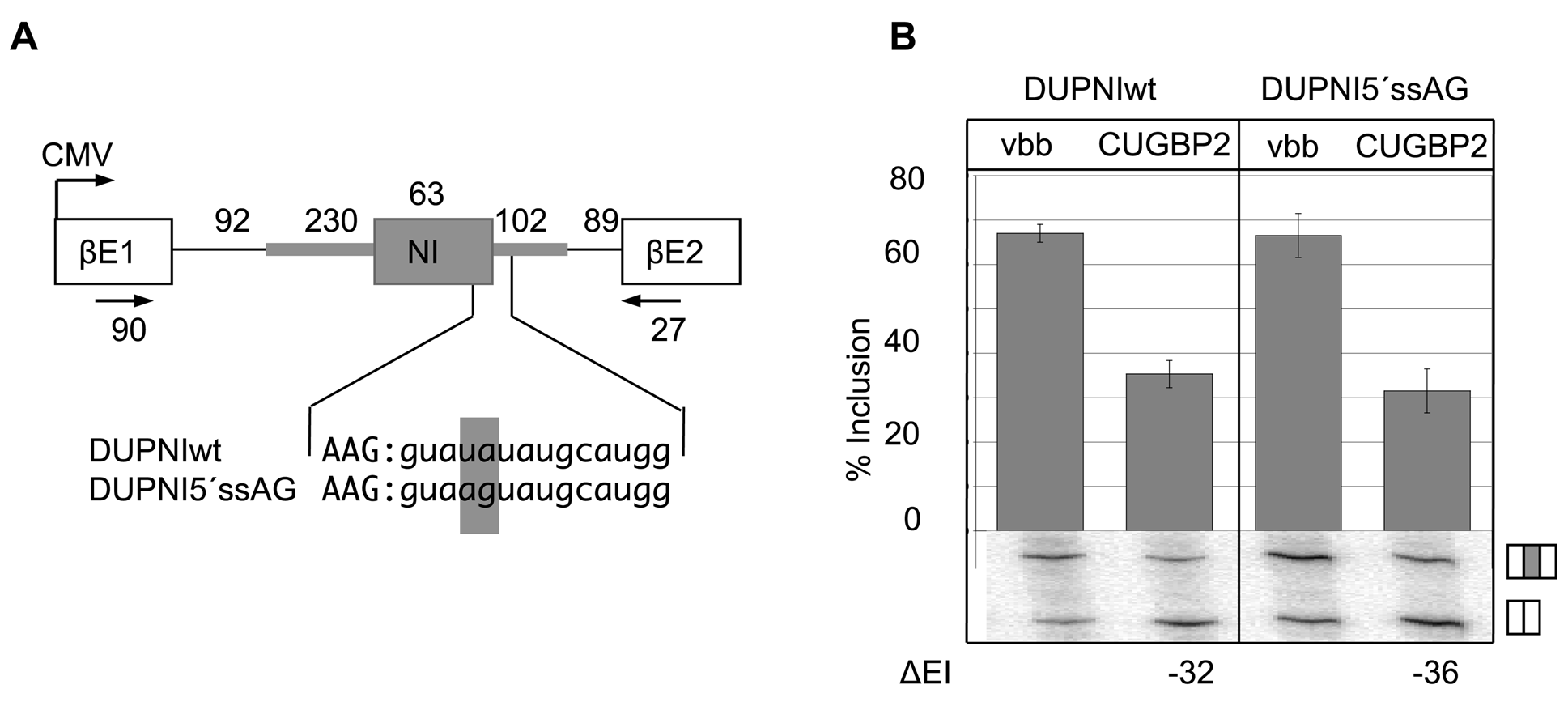

Supplement: Figure S6 — Strengthening the NI cassette exon 5′ splice site complementarity to U1 snRNP does not affect splicing silencing by CUGBP2. (A) Schematic of mutations made to the 5′ splice site to strengthen U1 snRNP binding (shaded nucleotides). (B) In vivo splicing assay with overexpression of a vector backbone control (vbb) or CUGBP2 protein expression vector (CUGBP2). Graph shows % exon inclusion values; error bars, standard deviations. (0.15 MB TIF) [file pgen.1000595.s006.tif]

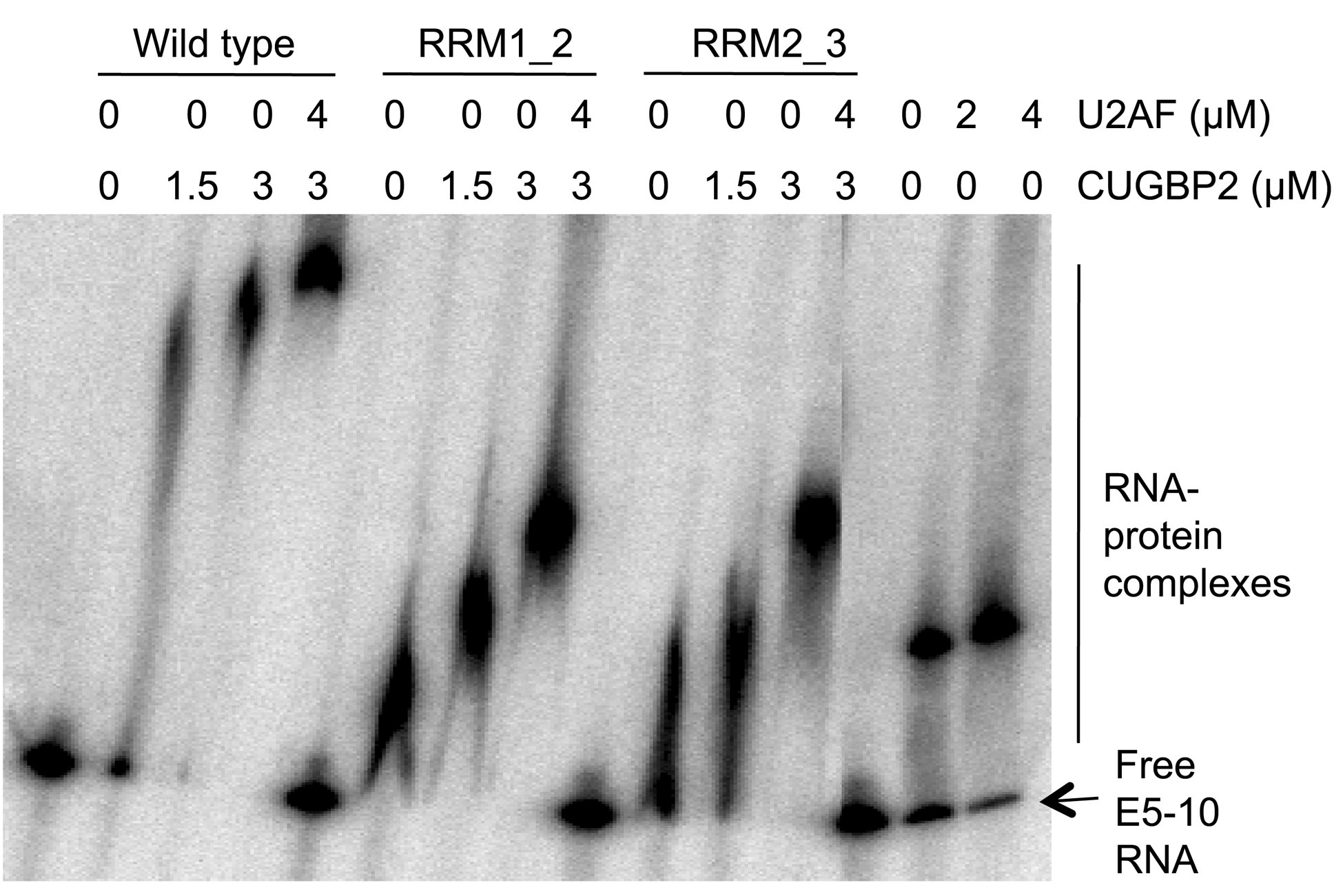

Supplement: Figure S7 — CUGBP2 and U2AF can bind to the same RNA at the same time. Increasing amounts of recombinant wild type or mutant CUGBP2 containing only RRMs 1 and 2 (RRM1_2) or RRMs 2 and 3 (RRM2_3) were bound to E5-10 RNA in the presence or absence of Hela purified U2AF. Protein concentrations are labeled on the top of the gel and free RNA and RNA-protein complexes are labeled at right. (0.70 MB TIF) [file pgen.1000595.s007.tif]
